# hAG-2 and hAG-3, human homologues of genes involved in differentiation, are associated with oestrogen receptor-positive breast tumours and interact with metastasis gene C4.4a and dystroglycan

**DOI:** 10.1038/sj.bjc.6600740

**Published:** 2003-02-18

**Authors:** G C Fletcher, S Patel, K Tyson, P J Adam, M Schenker, J A Loader, L Daviet, P Legrain, R Parekh, A L Harris, J A Terrett

**Affiliations:** 1 Oxford Glycosciences, 10 The Quadrant, Abingdon Science Park, Abingdon, Oxon, OX14 3YS, UK; 2 Hybrigenics, 3-5 Impasse Reille, 75014 Paris, France; 3 Cancer Research UK, Molecular Oncology Laboratories, Weatherall Institute of Molecular Medicine, John Radcliffe Hospital, Oxford OX3 9DS, UK

**Keywords:** hAG-2, hAG-3, breast tumours, oestrogen receptor, C4.4a, dystroglycan

## Abstract

hAG-2 and hAG-3 are recently discovered human homologues of the secreted *Xenopus laevis* proteins XAG-1/2 (AGR-1/2) that are expressed in the cement gland, an ectodermal organ in the head associated with anteroposterior fate determination during early development. Although the roles of hAG-2 and hAG-3 in mammalian cells are unknown, both proteins share a high degree of protein sequence homology and lie adjacent to one another on chromosome 7p21. hAG-2 mRNA expression has previously been demonstrated in oestrogen receptor (ER)-positive cell lines. In this study, we have used real-time quantitative RT – PCR analysis and immunohistochemistry on tissue microarrays to demonstrate concordant expression of hAG-2 and hAG-3 mRNA and protein in breast tumour tissues. Tumour expression of both genes correlated with OR (hAG2, *P*=0.0002; hAG-3, *P*=0.0012), and inversely correlated with epidermal growth factor receptor (EGFR) (*P*=0.003). Yeast two-hybrid cloning identified metastasis-associated GPI-anchored C4.4a protein and extracellular alpha-dystroglycan (DAG-1) as binding partners for both hAG-2 and hAG-3, which if replicated in clinical oncology would demonstrate a potential role in tumour metastasis through the regulation of receptor adhesion and functioning. hAG-2 and hAG-3 may therefore serve as useful molecular markers and/or potential therapeutic targets for hormone-responsive breast tumours.

Breast cancer is the most frequently diagnosed cancer in women and accounts for 30% of all cancers diagnosed in the United States (Greenlee *et al*, 2000). The implementation of screening programmes for the early detection of breast cancer, and the advent of anticancer treatments, such as chemotherapy, radiotherapy and antioestrogen therapies, to augment surgical resection have improved the survival of breast cancer patients. However, even breast tumours with good prognoses such as ER positive ductal carcinoma *in situ* (DCIS) become refractory to such treatments as the cancer cells develop resistance to chemotherapy drugs or lose their hormone sensitivity, leading to recurrent or metastatic disease that is often incurable. Thus, further characterisation of the molecular pathology of breast cancer (including hormone-responsive tumours) remains a key requirement in the development of better treatments.

In a previous proteomic analysis of purified membrane preparations from multiple human breast tumour-derived cell lines (Adam *et al*, in press) we identified a unique protein BCMP11. BCMP11 was homologous to hAG-2 (Genbank entry NM006408.2), an uncharacterised human protein encoded by a cDNA cloned from the MCF-7 breast cancer cell line (Thompson and Weigel, 1998) and localised to chromosomal band 7p21.3 (Petek *et al*, 2000). In view of the high degree of sequence identity (71%) between the two proteins, and the fact that they lie adjacent to one another at chromosomal position 7p21, we named BCMP11 as hAG-3 (Genbank entry AY069977). The functions of both hAG-2 and hAG-3 are unknown; however, they are related to the *Xenopus laevis* proteins XAG-1/AGR-1 and XAG-2/AGR-2. XAG-1 and XAG-2 are expressed in ectodermal cells during the development of the cement gland, a mucus-secreting organ in the head that is involved in the attachment of the frog embryo to a solid support (Sive *et al*, 1989). Indeed, the finding that XAG-2 is involved in the regulation of dorsoanterior ectodermal cell fate during cement gland differentiation (Aberger *et al*, 1998) suggests its potential role as a differentiation factor. hAG-2 and hAG-3 each have a predicted N-terminal cleavable secretory signal sequence (http://psort.nibb.ac.jp) and we previously demonstrated that hAG-3 was localised to endosomes in the T47D breast cancer cell line, further evidence that it is a secreted protein (Adam *et al*, in Press). hAG-2 was identified in a number of ER-positive breast cancer cell lines (Thompson and Weigel, 1998) and we previously demonstrated expression of hAG-3 immunohistochemically in breast cancer tissues (Adam *et al*, in Press).

Here we demonstrate using immunohistochemical and real-time quantitative RT–PCR analysis that hAG-2 and hAG-3 mRNA and protein exhibit a remarkably similar expression pattern in breast cancer tissues that strongly correlates with OR status and inversely with EGFR status. Furthermore, yeast two-hybrid cloning identified metastasis-associated GPI-anchored C4.4a protein and extracellular alpha-dystoglycan (DAG-1) as binding partners for both hAG-2 and hAG-3. These observations are intriguing since if they can be verified in clinical tumours they would suggest a potential role in breast tumour establishment and metastasis through secretion, cell and extracellular matrix (ECM) adhesion, and regulation of receptor function.

## MATERIALS AND METHODS

### Real-time quantitative RT–PCR

Real-time quantitative RT–PCR analysis of gene expression (Heid *et al*, 1996; Morrison *et al*, 1998) was carried out on first-strand cDNA derived from RNA isolated from samples of breast tumour tissues (provided by Prof AL Harris, University of Oxford, UK). All clinical samples were obtained with informed patient's consent and ethical approval. Each PCR reaction contained 10 ng first-strand cDNA (prepared from each mRNA sample using Superscript™ reverse transcriptase, Life Technologies, Carlsbad, CA, USA), SYBR green sequence detection reagents (Applied Biosystems, Foster City, CA, USA), and sense and antisense primers. All primer pairs traverse at least one intron and test products have been sequenced to confirm specificity before use in these assays. PCR products from all samples were analysed on agarose gels and positives shown to contain a single PCR product of the size predicted from cDNA. No fragments of the size predicted from genomic DNA were detected in any samples demonstrating the complete absence of genomic DNA contamination. All reactions were run twice and any samples showing a >10% variation in copy number excluded from analysis. The hAG-2 primers used were: F, agataccacagtcaaacctg (exon2): R, gcactcatccaagtgatgaa (exon4, inter-exon distance=652 bps) and hAG-3 primers were: F, ctggaggattgtcaatactc (exon3); R, gcataaggtttagcatgat(exon4, inter-exon distance=678 bps). The C4.4a primers used were: F, aagaatgaccgcggcctggatc (exon3); R, gacatgatcgctggcgttgtag (exon4, inter-exon distance=643 bps). The PCR conditions used for all sets of primers were 1 cycle at 95°C for 10 min, and 40 cycles at 95°C for 15 s, 57.5°C for 1 min. Reaction products were assayed on an ABI Prism 7700 sequence detection system (Applied Biosystems, Foster City, CA, USA) and the accumulation of PCR product was measured in real time as the increase in SYBR green fluorescence. Data were analysed using the Sequence Detector program v1.6.3 (Applied Biosystems, Foster City, CA, USA). Standard curves relating initial template copy number to fluorescence and amplification cycle were generated using the amplified PCR product as a template, and were used to calculate mRNA copy number in each sample. Data were expressed as copy number per nanogram cDNA.

### Immunohistochemistry

The hAG-2 polyclonal antibody was raised in rabbits immunised with two specific peptides (Abcam Ltd, Cambridge, UK). Peptide sequences were chosen for synthesis based on plots of hydrophobicity, antigenicity, surface probability, and minimal homology to other known protein family members. Peptides were synthesised using Fmoc chemistry with a cysteine residue added to the end of each to enable specific thiol-reactive coupling of Keyhole Limpet Haemocyanin prior to immunisation. The hAG-2 peptides used were VKPGAKKDTKDSRPK and LVYETTDKHLSPDGQ. The generation of the hAG-3 polyclonal antibodies has been described previously (Adam *et al*, in press). The ER monoclonal antibody (Dako Ltd., Glostrup, Denmark) is specific for the N-terminal region of ER*α*.

Immunohistochemical analysis was carried out on formalin-fixed paraffin-embedded tissue microarrays containing 1 mm sections of breast carcinoma tissues from 60 donors (obtained from Clinomics Laboratories Inc., Pittsfield, MA, USA). Slides were deparafinised by two 5 min washes in xylene, then rehydrated through successive graded ethanol solutions and washed for 5 min in PBS. Antigen retrieval was achieved by immersing the slides in 0.01 M citrate buffer (pH 6) and microwaving for 10 min at full power (950 W) and then treating the tissue with pepsin (1 mg ml^−1^) for 1.5 min at room temperature at pH 2. Endogenous hydrogen peroxidase activity was quenched by treating the slides in 3% hydrogen peroxidase/PBS for 10 min followed by two washes in PBS. The tissue was blocked in 10% donkey serum/PBS for 1 h before addition of 2 *μ*g ml^−1^ primary polyclonal antibody (in 2.5% donkey serum). Following three washes in PBS, the tissue sections were incubated with biotin-conjugated secondary antibodies (Biotin-SP-conjugated AffiniPure Donkey anti-rabbit, Jackson ImmunoResearch, West Grove, PA, USA) diluted at 1 : 200 (2.5 *μ*g ml^−1^ in 2.5% donkey serum/PBS) for 1 h. Slides were washed three times in PBS and the tissue incubated with streptavidin–HRP (Jackson ImmunoResearch, West Grove, PA, USA) diluted 1 : 100 (5 *μ*g ml^−1^ in 2.5% donkey serum/PBS), followed by three 5-min washes in PBS. Antibody signal was detected using DAB substrate solution (DAKO Ltd., Glostrup, Denmark) according to the manufacturer's instructions. Sections were screened for the presence of epithelial cells (Moll *et al*, 1982) using an anti-cytokeratin antibody (DAKO Ltd., Glostrup, Denmark) according to the manufacturer's instructions. Those sections without epithelial cells were not used.

### Yeast two-hybrid cloning analysis

Baits were PCR amplified (Pfu, Stratagene, La Jolla, CA, USA) and then cloned in the pB6 plasmid derived from the original pAS2ΔΔ (Fromont-Racine *et al*, 1997). PCR fragments were subcloned using classical enzymatic methods in a 96-well-plate format. All bait constructs were fully sequenced before transformation into yeast. The bait fragment used from hAG-2 was the predicted mature protein (amino acids 21–175 of Accession NP_006399) and did not contain the predicted cleaved signal sequence. An identical strategy was used for hAG-3 using the predicted mature protein (amino acids 24–166 of Accession AAL55402). Random and oligo dT-primed cDNA libraries from human placenta poly(A^+^) RNA and separately from poly(A^+^) RNA pooled from T47D, MCF7, MB-MDA-468, and BT20 breast cancer-derived cell lines were constructed into the pP6 plasmid derived from the original pACT2 plasmid (Rain *et al*, 2001) and transformed in Escherichia coli (DH10B; Invitrogen, Carlsbad, CA, USA). The complexity of the primary libraries was over 50 million clones. Sequence analysis was performed on 300 randomly chosen clones to establish the general characteristics of each library. The libraries were then transformed into yeast and 10 million independent yeast colonies were collected, pooled, and stored at −80°C as equivalent aliquot fractions of the same library.

The mating protocol has been described elsewhere (Fromont-Racine *et al*, 1997). Briefly, the screening conditions were adapted for each bait (test screen) before performing the full-size screening. The selectivity of the *HIS3* reporter gene was modulated with 3-aminotriazole in order to obtain a maximum of 384 histidine-positive clones. For all the selected clones, *LacZ* activity was measured in a semiquantitative X-Gal overlay assay. The interacting ‘prey’ fragments of the positive clones were amplified by PCR, analysed on an agarose gel, and sequenced at their 5′ and 3′ junctions on a PE3700 Sequencer. The resulting sequences were then used to identify the corresponding gene in the GenBank database (NCBI).

## RESULTS

### hAG-3 and hAG-2 share a high degree of sequence homology and lie adjacent to one another at chromosomal position 7p21

Figure 1A

**Figure 1:**
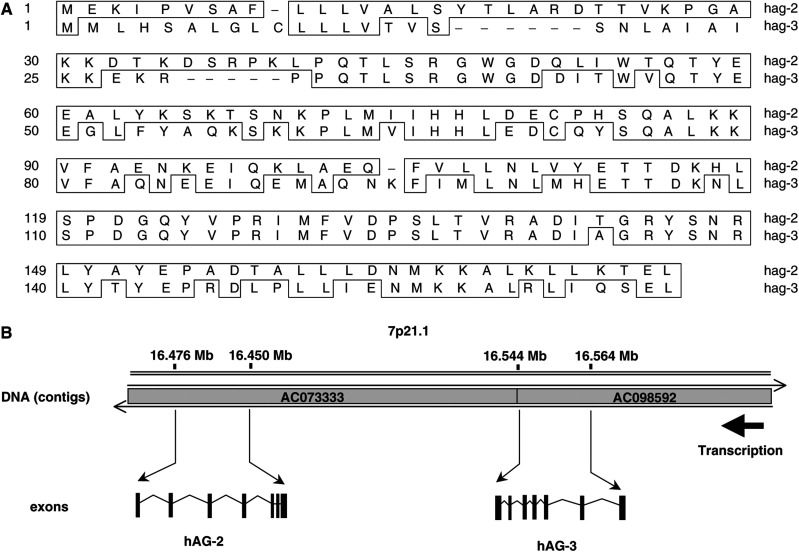
hAG-2 and hAG-3 show close homology at the protein level and are adjacent to each other at chromosome position 7p21. (**A**) Protein sequence alignment of hAG-2 and hAG-3 (DNAStar MegAlign program–Jotun Hein alignment). The boxed regions on the hAG-3 sequence indicate exact amino-acid matches of hAG-3 with hAG-2. (**B**) Diagram illustrating the genomic locations, transcriptional orientation, and exon structures of hAG-2 and hAG-3 on chromosome 7p21.

shows the protein sequence alignment of hAG-3 with hAG-2. Overall, both proteins show 71% similarity (Jotun-Hein method, 64% identity by BLASTP) with the majority of differences being in the hydrophobic leader signal sequences. A BLAST (Altschul *et al*, 1990) search of the ENSEMBL genomic database (http://www.ensembl.org) with the hAG-3 coding sequence localised the gene to chromosome 7p21 (Figure 1B). The hAG-3 sequence is not linked to hAG-1, which lies on chromosome 1, but is in the same genomic segment as hAG-2 (Petek *et al*, 2000), transcribed from the same DNA strand and separated by approximately 60 kb of genomic DNA. Both hAG-2 and hAG-3 genes comprise seven coding exons with similar exon/intron boundaries, which together with the high similarity and close genomic locality suggest that they arose from a gene duplication event.

### Coexpression of hAG-2 and hAG-3 mRNA and protein in ER-positive breast cancer tissues

**Figure 2:**
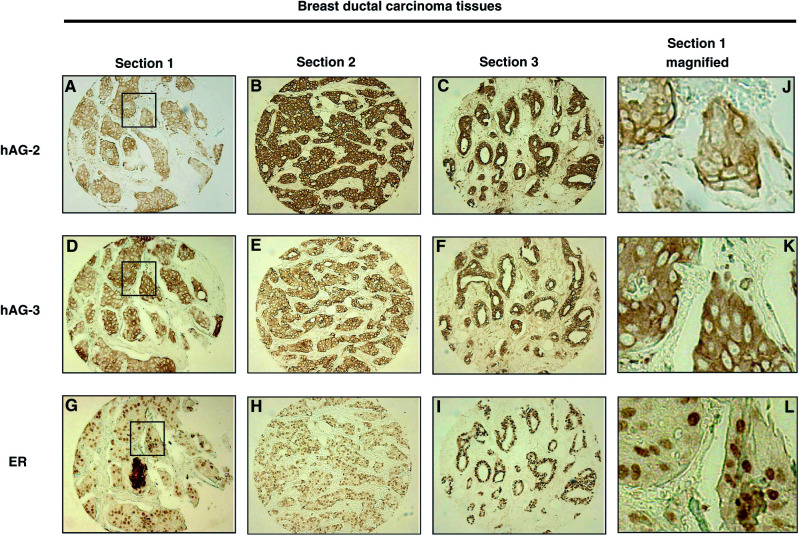
Immunohistochemical analysis of hAG-2, hAG-3, and ER protein in three separate breast ductal carcinoma donor tissue sections. The boxed region in panels **A**, **D**, and **G** is magnified in panels **J**, **K**, and **L**, demonstrating cytoplasmic staining of hAG-2 (**J**) and hAG-3 (**K**) and nuclear staining of ER (**L**).

Immunohistochemical analyses demonstrated that hAG-2, hAG-3, and ER protein expression was detected in 48 (83%), 43 (74%), and 34 (59%), respectively, of 58 breast cancer sections. hAG-2 and hAG-3 immunostaining was restricted to the cancerous epithelial cells of the tumour tissue and was predominantly cytoplasmic (Figure 2

). Coexpression of hAG-2 and hAG-3 in the breast tumour tissues was highly significant (*P*<0.0001). Expression of both hAG-2 and hAG-3 also strongly correlated with OR expression (hAG-2, *P*=0.0002; hAG-3, *P*=0.001), examples of breast tumour sections showing immunostaining for all three proteins are shown in Figure 2. Nevertheless, there were a number of ER-negative breast cancers that expressed both hAG-2 and hAG-3.

For a more quantitative assessment of hAG-2 and hAG-3 levels in breast tumour tissues and their association with several biological parameters, we performed real-time quantitative RT–PCR on 46 cDNA samples derived from breast tumour tissues. In all, 23 of these tissues were derived from patients with lymph node metastasis, the remaining 23 showed no lymph node metastasis. The results of these analyses are shown in (Table 1

**Table 1:** Expression of hAG-3 and hAG-2 mRNA in 46 breast cancer samples

**Gene**	**Biological marker**	**mRNA copy number median (range)**	**Statistical significance**
hAG-2	ER+	13000 (2900–160 000)	*P*=0.01
	ER−	4500 (130–78 000)	
hAG-3	ER+	5774 (88–30 573)	*P*=0.004
	ER−	183 (5–16 961)	
hAG-2	Age⩾50 years	11 000 (130–160 000)	*P*=0.75
	Age<50 years	8900 (600–130 000)	
hAG-3	Age⩾50 years	3738 (12–30 573)	*P*=0.05
	Age<50 years	903 (5–6608)	
hAG-2	EGFR−	16 000 (130–160 000)	*P*=0.009
	EGFR+	5650 (290–17 000)	
hAG-3	EGFR−	5774 (63–30 573)	*P*=0.003
	EGFR+	233 (5–13 216)	
hAG-2	Tumour Grade I, II	12 500 (150–160 000)	*P*=0.09
	Tumour Grade III	4950 (130–130 000)	
hAG-3	Tumour Grade I, II	6295 (282–30 573)	*P*=0.006
	Tumour Grade III	183 (5–16 961)	

hAG-2 and hAG-3 expression in 46 clinical breast cancer samples was quantified by real time RT-PCR. mRNA copy number per ng cDNA was correlated with ER status (+, positive: −negative), age (less than 50 years of age versus 50 years of age and over), EGFR status, and tumour grade (grades I and II *vs* grade III). Statistical significance was calculated using the Mann Whitney U-test.

). Both hAG-2 and hAG-3 showed a correlation with ER expression and a negative correlation with EGFR expression (Table 1). Expression of hAG-3, but not hAG-2, was associated with tumour grades I/II and patients aged⩾50 years (Table 1). Moreover, there was no significant association of either hAG-2 or hAG-3 expression in the primary tumours with the presence of lymph node metastasis (data not shown). Although there was high concordance with OR-positive primary breast cancers, OR expression is not associated with node metastasis either.

**Figure 3:**
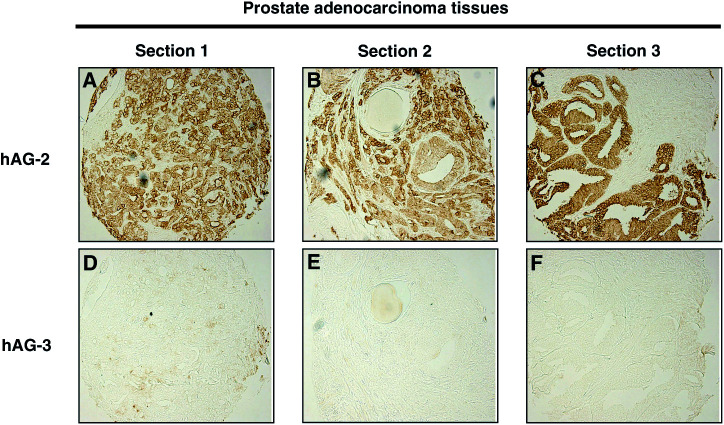
hAG-2, but not hAG-3, protein is expressed in malignant prostate epithelial cells. Immunohistochemical analysis of hAG-2 and hAG-3 in three prostate adenocarcinoma donor tissue sections.

In contrast to the breast tumour data, hAG-2 and hAG-3 were not coexpressed in another hormone-responsive tumour type, prostate cancer. Of 42 prostate cancer sections examined by immunohistochemistry, 34 (81%) were immunoreactive for hAG-2 and only 7 (17%) immunoreactive for hAG-3. Examples of hAG-2 and hAG-3 immunostaining in the same donor prostate tumour sections are demonstrated in Figure 3

.

### Both hAG-2 and hAG-3 proteins interact with metastasis-associated C4.4a protein and alpha-dystroglycan (DAG-1)

**Figure 4:**
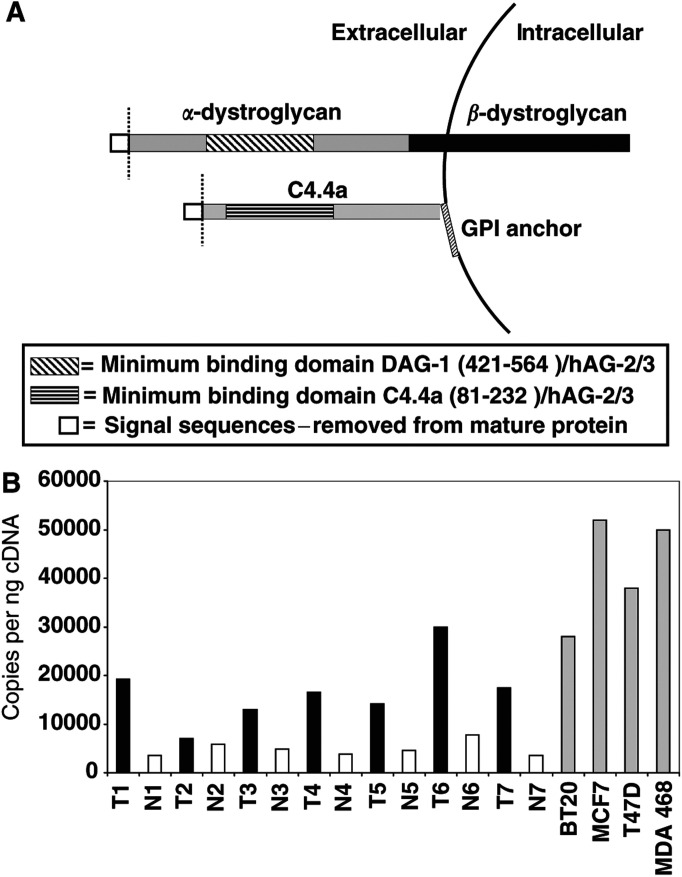
(**A**) Diagrammatic illustration of DAG-1 and C4.4a proteins showing the extracellular hAG-2 and hAG-3 minimum binding domains identified by yeast two-hybrid analyses. Although both structures are shown attached to the same cell, it is likely that the interactions of the hAG proteins are autocrine in nature for C4.4a and paracrine for DAG-1. (**B**) Real-time quantitative RT–PCR analysis of C4.4a mRNA expression in seven donor-matched adjacent normal (N) and tumour (T) breast samples (indicated 1–7) and four human breast cancer-derived cell lines. C4.4a is upregulated in all of the tumour samples, relative to their matched normal tissue, and expressed in all the breast cancer cell lines used to create the yeast two-hybrid prey library. Expression values are described as copies per nanogram of cDNA derived from a standard curve of known dilutions of C4.4a DNA.

The similarities between hAG-2 and hAG-3 and their significance to breast cancer are further characterised by the interacting proteins identified for hAG-2 and hAG-3 in a yeast two-hybrid screen. We have already shown that hAG-3 interacts with the human homologue of a rat GPI-anchored protein C4.4a (GenBank: NM_014400) (Adam *et al*, in Press). Analysis of hAG-2 and hAG-3 bait proteins (with their signal sequences deleted) in the same yeast two-hybrid system (Rain *et al*, 2001) using the same placenta-derived prey library as well as a prey library derived from a pool of four breast cancer cell lines (T47D, MCF7, BT20, MDMBA468) identified C4.4a and dystroglycan (DAG1, GenBank Accession XP_018223) as the only significant interacting partners for both proteins. The significance of these interactions from screening two prey libraries is defined by: (1) neither DAG-1 nor C4.4a have been identified as binding partners in screens of more than 250 other proteins in this yeast two-hybrid system (Daviet L, unpublished observation), (2) both DAG-1 and C4.4a are identified as binding partners for hAG-2 and hAG-3 from the placenta and breast cancer prey libraries, (3) multiple clones for both DAG-1 (43) and C4.4a (8) with different but overlapping interaction domains were observed for both hAG-2 and hAG-3 allowing minimum binding domains to be defined for DAG-1 and C4.4a, and (4) these minimum binding domains (Figure 4A

) were restricted to extracellular portions of DAG-1 (alpha dystroglycan chain) and C4.4a consistent with the predicted secreted nature of the hAG proteins.

Real-time quantitative RT–PCR analysis demonstrated that C4.4a mRNA expression is elevated in seven clinical breast tumours compared with the matched adjacent normal breast tissue. In addition, C4.4a is also highly expressed in the four human breast cancer-derived cell lines used for the construction of the yeast two-hybrid library (Figure 4B). Clearly, further analyses such as coimmunoprecipitation are required to confirm that these interactions occur in clinical cancers but the consistency of the yeast 2-hybrid data, the expression profile of C4.4a and the previously reported links of DAG-1 and C4.4a to cancer make these interactions intriguing enough to warrant further investigation.

## DISCUSSION

We have characterised the expression profile and protein binding partners of the two homologous, functionally uncharacterised proteins, hAG-2 and hAG-3. Both genes lie adjacent to one another at chromosomal position 7p21, are 71% identical and are concordantly expressed in OR-positive breast tumour tissues. Both hAG-2 and hAG-3 have also been shown to interact with the same two proteins, C4.4a and alpha dystroglycan, in yeast two-hybrid cloning assays using two different libraries.

The coexpression of hAG-2 and hAG-3 with oestrogen receptor is intriguing. Indeed a search of the first 20 kb of both the hAG-2 and hAG-3 promoters has identified four and 12 putative oestrogen response elements, respectively (using an consensus sequence of GGTCAnnnTGACC) (Schenker M, unpublished observations). Despite the striking correlation of hAG-2 and hAG-3 with OR, over half of the OR-negative breast tumours examined immunohistochemically expressed hAG-2 and hAG-3, suggesting a more penetrant role for both proteins in breast cancer aetiology than is defined by OR alone. This suggests that both hAG-2 and hAG-3 genes may be transcriptionally regulated by factors in addition to OR. Indeed, the complete lack of correlation between hAG-2 and hAG-3 expression in another hormone-responsive cancer, namely prostate cancer, clearly indicates differences in the transcriptional regulation of both genes.

The finding that hAG-2 and hAG-3, two unique secreted proteins whose expression is elevated in OR-responsive breast tumours, interact with the same two known extracellular proteins *in vitro*, C4.4a and dystroglycan, could indicate a role for the hAG proteins in the development and/or progression of breast cancer consistent with the known developmental role of AGR1/2 in Xenopus embryos. Apart from its reported association with metastasis (Rosel *et al*, 1998), C4.4a gene expression has recently been shown to be elevated in urothelial cells exposed to matrigel *in vitro*, a model designed to discover proteins involved in cell–cell and cell–matrix interactions (Smith *et al*, 2001). Thus, the observed yeast 2-hybrid interactions of both hAG proteins with C4.4a could associate these secreted proteins with a GPI-anchored receptor protein involved in hormone responsiveness, cell adhesion, migration, and metastasis. As such, the hAG/C4.4a inter-action may represent a viable target for oestrogen-responsive breast cancer intervention. Moreover, our finding that C4.4a mRNA expression is upregulated in breast cancer tissues compared with adjacent normal tissues, and significantly expressed in a number of breast cancer cell lines, further supports a clinically important role for the association of the hAG proteins with C4.4a.

The minimum binding domain of dystroglycan (DAG-1) for both hAG-2 and hAG-3 is shown in Figure 4A. This sequence corresponds to the extracellular alpha dystroglycan chain of DAG-1. The complete DAG-1 protein compromises two subunits, 43 kDa transmembrane and 156 kDa extracellular, derived from the same mRNA, and provides a link between the sarcolemma and extracellular matrix through the binding of laminin (Ibraghimov-Beskrovnaya *et al*, 1992). This provides further evidence that hAG-2 and hAG-3 are secreted proteins and associated with the ECM. Furthermore, DAG-1 interacts with and regulates caveolin-3 distribution which in turn affects alpha-integrin 7 receptor expression (Sotgia *et al*, 2000; Cote *et al*, 2002) so that the hAG/DAG-1 interaction could be affecting a further receptor pathway. Alpha dystroglycan is almost undetectable in cancer cell lines by Western blotting (Losasso *et al*, 2000), and this is reflected by the low number of clones (2) obtained from the human breast cancer cell line prey library compared with the placenta library (40). The hAG/DAG-1 interaction could therefore have more of a paracrine function than the hAG/C4.4a interaction, and thus be involved in cell–cell interactions between cancer and noncancer cells and the intervening extracellular matrix.

In summary, two of the three known members of the hAG family of proteins show clear relevance to breast cancer that is strengthened by their roles in Xenopus development, a significant association with ER, and putative protein-binding partners that are not only linked with cancer progression but provide opportunities for those interactions to be targeted in the development of cancer therapies.
